# Analysis and modification of defective surface aggregates on PCDTBT:PCBM solar cell blends using combined Kelvin probe, conductive and bimodal atomic force microscopy

**DOI:** 10.3762/bjnano.8.62

**Published:** 2017-03-08

**Authors:** Hanaul Noh, Alfredo J Diaz, Santiago D Solares

**Affiliations:** 1 Department of Mechanical and Aerospace Engineering, The George Washington University, Washington, DC 20052, United States of America

**Keywords:** conductive atomic force microscopy, Kelvin probe force microscopy, multifrequency AFM, organic photovoltaics, polymer solar cells, surface defects

## Abstract

Organic photovoltaic systems comprising donor polymers and acceptor fullerene derivatives are attractive for inexpensive energy harvesting. Extensive research on polymer solar cells has provided insight into the factors governing device-level efficiency and stability. However, the detailed investigation of nanoscale structures is still challenging. Here we demonstrate the analysis and modification of unidentified surface aggregates. The aggregates are characterized electrically by Kelvin probe force microscopy and conductive atomic force microscopy (C-AFM), whereby the correlation between local electrical potential and current confirms a defective charge transport. Bimodal AFM modification confirms that the aggregates exist on top of the solar cell structure, and is used to remove them and to reveal the underlying active layer. The systematic analysis of the surface aggregates suggests that the structure consists of PCBM molecules.

## Introduction

Polymer solar cells (PSCs) [1] have been widely studied due to the abundance of their constituents, their mechanical flexibility and light weight, as well as the possibility of low-cost roll-to-roll mass production [2]. There have been immense improvements regarding the efficiency of photovoltaic devices [3], their lifetime [4] and their stability [5], such that recent PSCs show power conversion efficiencies (PCE) above 10% [6] and an intrinsic lifetime approaching seven years [4]. Since the majority of modern PSCs consist of nanoscale bulk heterojunction (BHJ) structures [7], changes in the internal structures, such as local crystallization of polymers or phase separation of donor and acceptor mixtures, greatly affect the device performance [8–9]. Extensive research has been performed to optimize the morphology of the active layer by controlling fabrication parameters such as donor/acceptor ratio, casting solvents, additives, and thermal and solvent annealing processes [8,10–12].

In addition to device-level investigations, studies on the local morphology changes of photoactive layers have continuously attracted much interest [13–14]. Due to the complicated nanoscale three-dimensional distribution of donor and acceptor materials in BHJ structures, atomic force microscopy (AFM) has been a very useful tool to observe the dependence of performance on the local morphology of PSCs [15], thus providing insights into the operating mechanism of PSCs. Among all the available modes of AFM, conductive AFM (C-AFM) and Kelvin probe force microscopy (KPFM) are the most widely used for PSC research, since the measured current and electrical potential can reveal local charge transport characteristics and the distribution of carriers and materials, which are relevant to device performance [16].

In this work, we use a series of AFM techniques to characterize electrically defective surface structures aggregated on test PSC specimens. The active layer of the test PSCs comprises the donor and acceptor materials poly[*N*-9′-heptadecanyl-2,7-carbazole-*alt*-5,5-(4′,7′-di-2-thienyl-2′,1′,3′-benzothiadiazole)] (PCDTBT) [17] and [6,6]-phenyl C71-butyric acid methyl ester (PCBM), respectively. The surface aggregates on the blend exhibit different frequencies of occurrence, depending on the solvent used in the fabrication process (chlorobenzene or dichlorobenzene). We characterize the spatial and electrical properties of the aggregates, and show that the structures are clusters of thin molecular layers exhibiting hole-blocking properties, which can affect charge transport efficiency at the blend–electrode interface. In addition, we use bimodal AFM, a multifrequency AFM technique [18], to modify and remove the surface molecular layers. The series of AFM analyses and modification performed can be useful to better understand the nature of PSC surface defects. A detailed description of the setup and methods is provided in Figure S1 (Supporting Information File 1) and in the Experimental section below.

## Results and Discussion

### Appearance of surface aggregates on the blend

In order to investigate the effect of casting solvent on the PSC morphology, we prepared test samples using either dichlorobenzene (DCB) or chlorobenzene (CB) as solvent. As described in the Experimental section, PCDTBT (the donor polymer) does not fully dissolve in CB, whereas it is almost completely dissolved in DCB. As a consequence, the CB mixture contains precipitated and dissolved PCDTBT components. Because CB samples are fabricated only with the dissolved portion, they are expected to contain a high percentage of short-chain PCDTBT, which has a relatively small molecular weight [19]. On the other hand, samples processed with DCB have all the components of PCDTBT. Besides the ratio of short- to long-chain polymers, the solubility differences of donor and acceptor materials and the boiling point of the solvents affect the morphology and thus the performance of PSCs [20]. The PCDTBT:PCBM blend fabricated with the DCB solvent has shown better performance than the one cast from CB [21].

**Figure 1:**
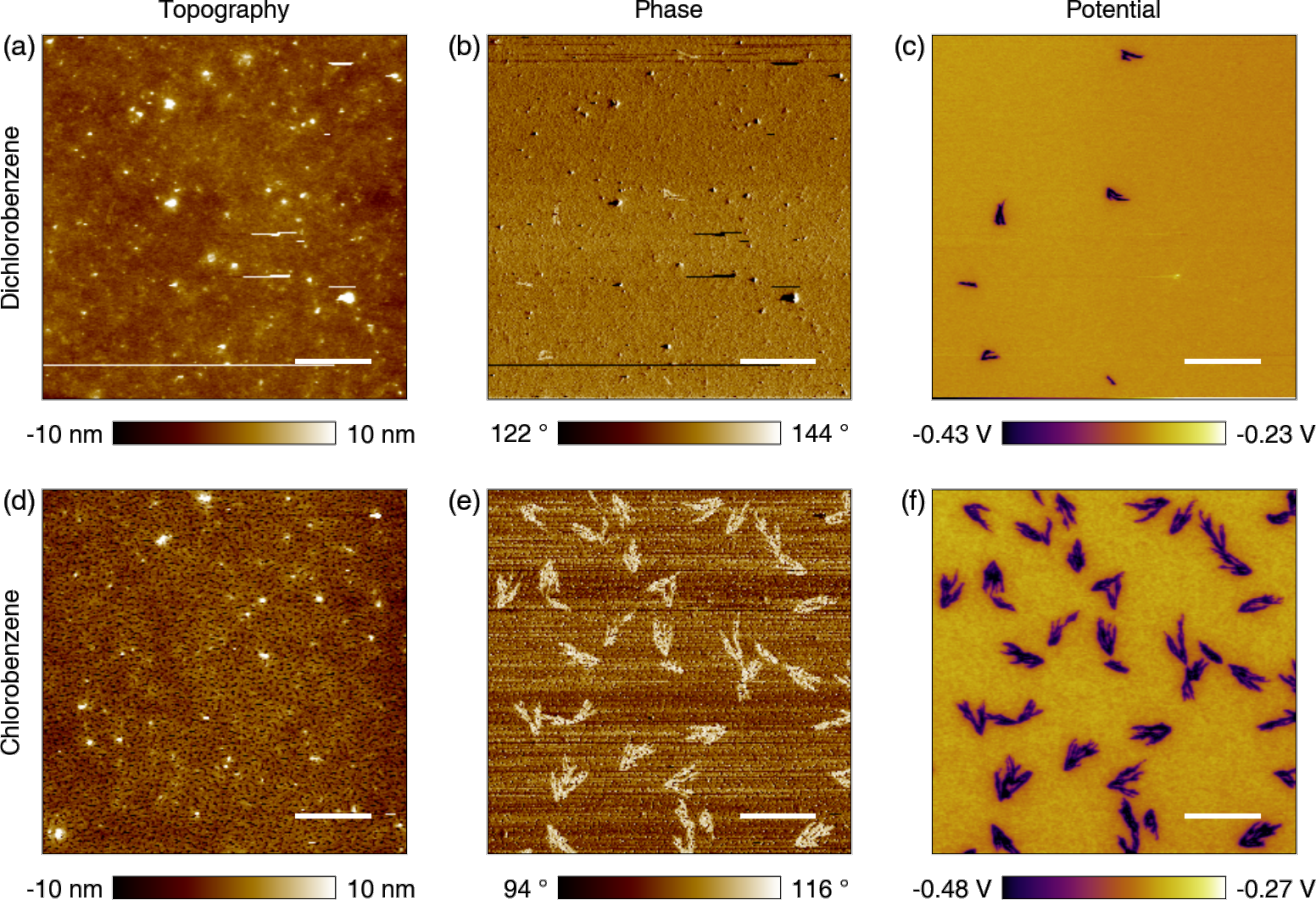
KPFM surface potential measurements of samples cast from DCB and CB. (a) Topography of the sample fabricated with DCB. (b) Corresponding phase in amplitude-modulation AFM. (c) Potential showing dark features estimated at 0.76% of the entire scan area. (d) Topography of the sample fabricated with CB. (e) Corresponding phase showing the features as bright spots. (f) Potential showing the features, estimated at 15% of the entire scan area. The scale bars are 10 μm.

We performed KPFM measurements on both the DCB- and CB-cast samples to examine possible surface potential changes due to morphology differences of the PSC active layers. As shown in Figure 1, a clear difference in surface potential is observed, although there is no obvious correlation with the topography channel. The population of abnormal features in the potential measurement is much larger for the CB sample, which has 20 times more featured (dark) area in potential than the DCB case for the measured sites, whereby the dark areas are calculated as 15% and 0.76% of the entire scan area (50 × 50 μm^2^) for CB and DCB, respectively. A similar trend is observed for various other scan sites and samples. It is worth noting that the scan size has to be large enough for the DCB samples in order to find the structures of interest, whereas it is not difficult to find the structures in the CB samples within a scan size of 20 × 20 μm^2^. To study the abnormal features, we used the CB cast samples in the rest of our study.

**Figure 2:**
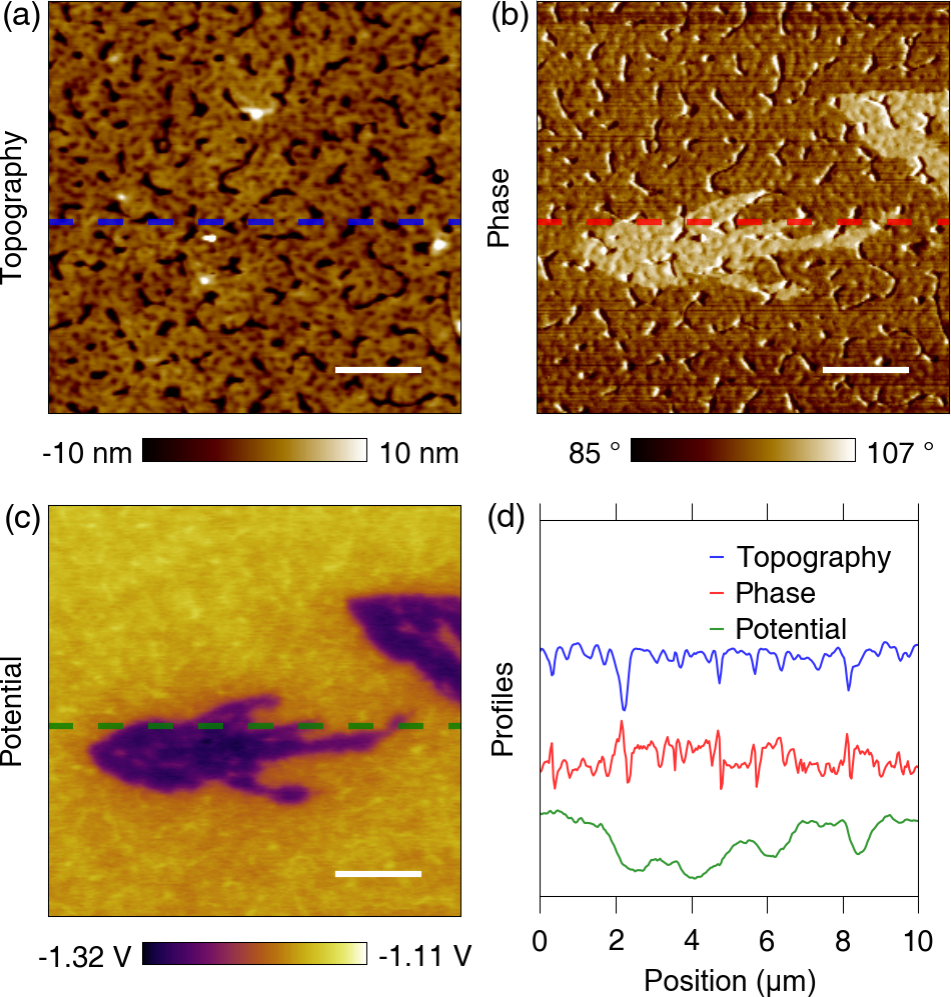
KPFM measurement of a typical feature in the CB-cast sample. (a) Topography showing no correlation to the features observed in the other signals. (b) Phase showing elevated signals for the features of interest. (c) Potential revealing the corresponding variations observed in the phase image. (d) Line profiles of topography, phase and potential along the dashed lines in the images. The scale bars are 2 μm.

In Figure 2, enlarged images of one of the features in the CB cast sample are shown. One can easily find particular regions that exhibit contrast against the ordinary surroundings in the phase (Figure 1e and Figure 2b) and potential (Figures 1f and 2c). The potential drops at the outlier region and the phase exhibits corresponding step-like variations. The corresponding topography in Figures 1d and 2a shows no observable structures. However, we have observed that the topography exhibits different behavior for fresh and aged samples. For the fresh samples used for the measurements of Figure 2, the images do not typically show any correlation between the measured topography and the features of interest in the phase and potential. If the imaging parameters are properly set, cross-talk artifacts between electrostatic [22] and van der Waals forces and topography can be avoided.

On the other hand, aged samples stored for several weeks under ambient conditions show topographical features corresponding to the structures found in the phase and potential measurements (see Figure S3, Supporting Information File 1). This observation suggests that there could be additional morphology changes after the fabrication process has been completed. These changes might be related to degradation processes and/or a slow evaporation of the remaining solvent over time. We also observed evolution of the features over time, starting right after manufacturing of a fresh sample (see Figure S4, Supporting Information File 1). The growth of island-like structures has been reported previously [23]. Although there are differences regarding the donor material used, the methods followed and the size of the island-like features, the authors of this previous study reported that PCBM crystallites are formed and grow in size according to the processing time of solvent vapor annealing (slow drying of a film) [23]. Since the degradation and/or the evaporation of remaining solvent from bare active layers will be different from those of samples buried under a top electrode and an encapsulation layer, we mostly used fresh samples to evaluate the properties of the features in order to avoid additional uncertainty (any deviations from this approach are noted in the text).

In order to further characterize the features, we analyze the topography changes on the aged sample. The features in this sample exhibit an interesting behavior. They gradually disappear over consecutive KPFM scans (see Figure S5, Supporting Information File 1). This phenomenon only happens for the aged samples, whereas fresh samples do not change over time. Furthermore, it only occurs during KPFM measurements, where the tip is biased with DC and AC voltages, and does not occur during basic AFM imaging where there are no bias voltages. Since KPFM was operated in the non-contact regime, i.e., with the tip dynamics influenced by attractive interactions, we expect that the removal of features is not due to direct contact between the tip and the features. We rather speculate that the features of interest are initially located on top of the active layer, subsequently removed from the sample, and finally attached to the tip due to attractive interaction between the tip and feature material. Although the source of the attractive forces is not well understood, it could be attributed to either electrostatic forces due to localized charges in the features or to electrophoresis of the material due to the electric field gradient.

**Figure 3:**
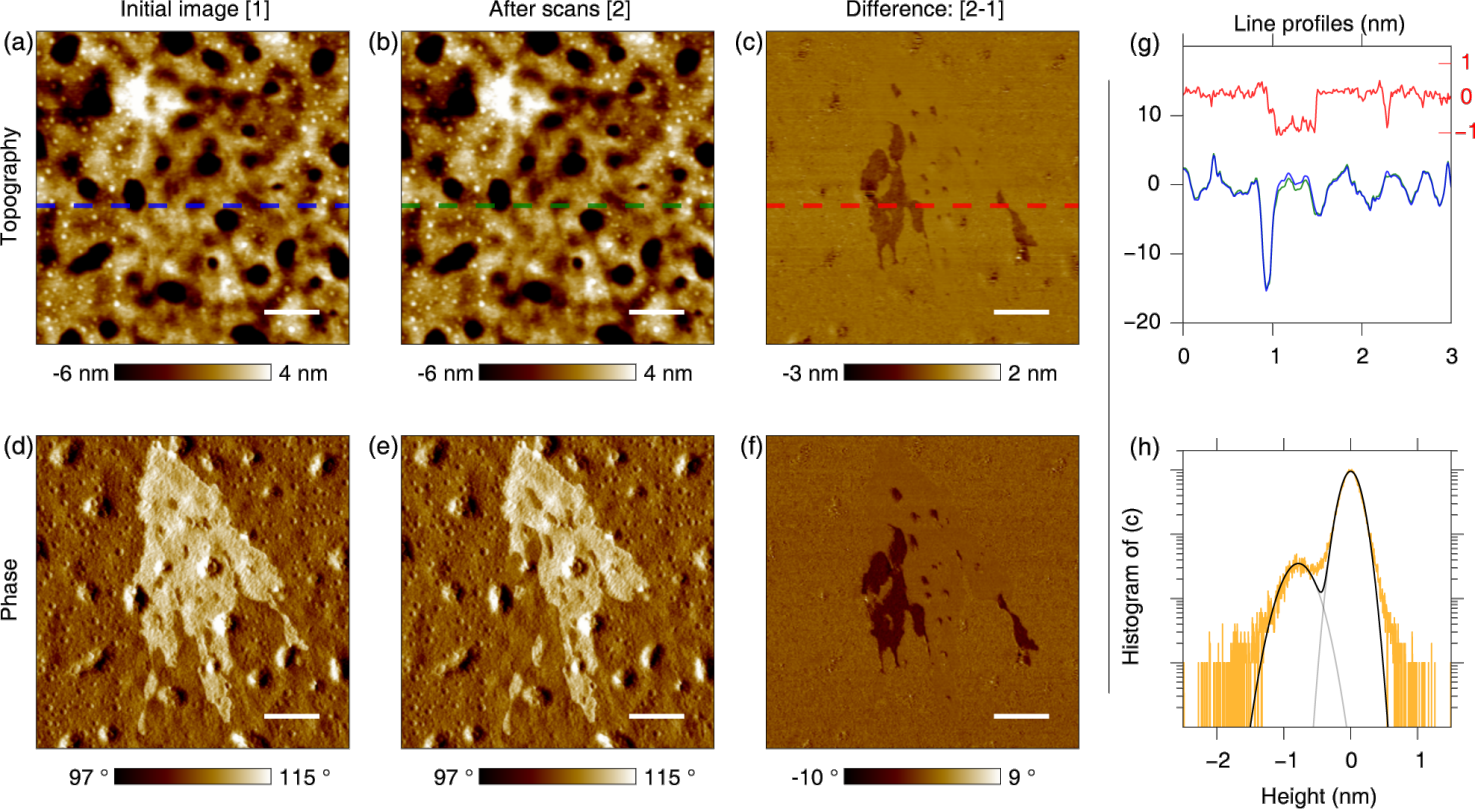
Removal of the surface aggregates in the aged sample. (a) Topography of initial sample. (b) Topography after several scans. (c) Difference between the two topographies showing clear changes. (d) Phase of the initial sample. (e) Phase after several scans. (f) Difference of the two phase images showing a similar change as the topography. (g) Line profiles of the initial (blue) and final (green) topography, and the difference (red, with a zoomed-in axis on the right) between the two topographies along the dashed lines in a–c. (h) Histogram (yellow) of the topography difference showing a height difference of ca. 0.8 nm between the two Gaussian peaks (black). The scale bars are 500 nm.

In Figure 3, a set of first- and last-scan images for the aged sample are shown. Although it is very difficult to distinguish the changes in topography, one can easily discern the partial removal of the brighter area from the phase images. In addition, the changed region can be clearly seen after calculating the difference between the two topography images (Figure 3c). Figure 3g shows the line profiles of the dashed lines in Figure 3a–c (blue, green and red for initial, last and difference image profiles, respectively). The height difference of the changed region (gap area between the blue and green curves) is relatively small compared to the overall profile. To calculate the height variation for the removed area, a histogram of the topography difference was obtained and fitted with a double Gaussian curve (Figure 3h). The measured height difference was ca. 0.8 nm. Based on this, we attribute the features to thin molecular layers formed on top of the bulk film. Although an artifact based on electrostatic interactions could also influence the measured height, we can exclude this possibility because the electrostatic force is a long-range force [24] that leads to blurred boundaries and lower resolution than in the phase images, as is clear in the potential images. On the other hand, the phase images exhibit high resolution and clear boundaries for the features, as well as step-like constantly elevated contrast. This means that the phase changes are primarily caused by interactions different from electrostatic force variations. The featured structure seems to be a cluster of aggregated layer portions, as supported by the group exfoliation behavior shown in Figure S5 (Supporting Information File 1).

It is worth noting here that the existence of ultra-thin mesoscopic clusters has not yet been reported, despite numerous studies on PCDTBT-PCBM. On the other hand, it has been shown that chromosome, sheaf-like PCBM crystals can grow within PCDTBT [25]. Surface stratification with the formation of an almost pure top-surface-enriched PCBM layer has also been reported for PCDTBT-PCBM [26]. In previous reports, it has been shown that compositional variation occurs in the entire volume of the active layer. However, since SPM methods primarily characterize the surface (with or without tip–sample contact), we conclude that the ultra-thin layers investigated in this report rest on the surface. We also point out that the samples used here are not optimized for typical PSC operating conditions (e.g., thermally annealed or solvent-treated for maximum conversion efficiency), so there may be a possibility to observe structures that have not been previously reported or investigated.

### Electric property mapping of surface aggregates

**Figure 4:**
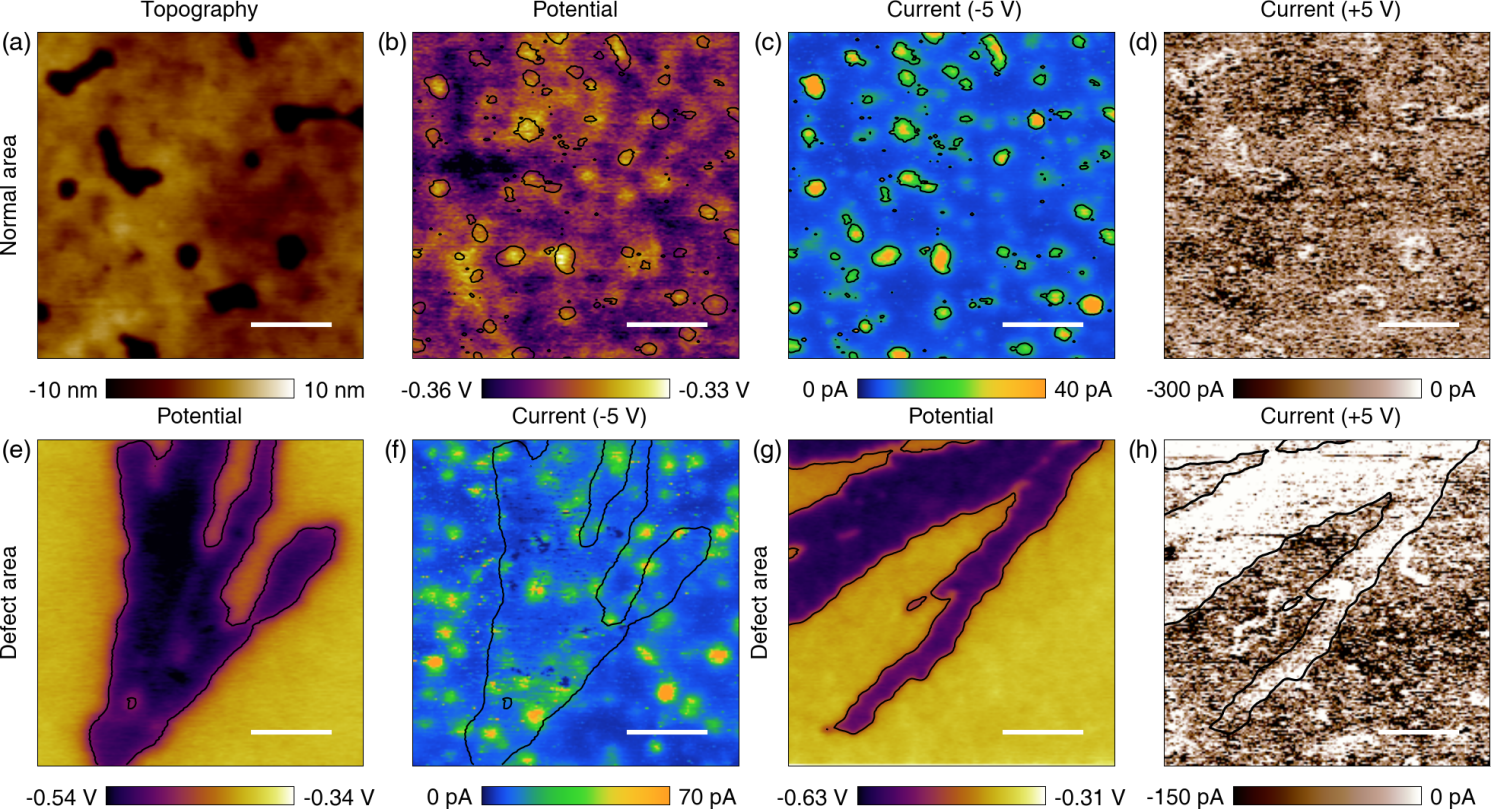
Correlation of the potential and current for normal and defective areas. (a) Topography of a normal bulk area. (b) Potential of the normal area. (c) Current for the same area measured with forward (−5 V) tip bias. (d) Current of the same area for reverse (+5 V) tip bias. (e) Potential for a defective area containing surface aggregates. (f) Corresponding forward current. (g) Potential for a second defective area. (h) Corresponding reverse current. Black contour lines highlight the correlation between potential and electron current in (b) and (c ) and the defect boundaries in panels (e)–(h). The scale bars are 500 nm.

We investigated the electric properties of the surface aggregates by performing and correlating KPFM and C-AFM at the same positions on randomly chosen areas. Topography, potential, forward current (−5 V tip bias with respect to the ITO substrate) and reverse current (+5 V tip bias) were sequentially obtained for a normal bulk area (containing no aggregates) and for areas containing surface aggregates, as shown in Figure 4. For the normal area, the measured potential shows a small peak-to-peak change of ca. 30 mV, whereas the aggregated areas typically exhibit a potential that is ca. 0.1–0.3 V lower than that of their surroundings. The measured forward and reverse currents for the normal areas show different distributions, similar to a previous report [27]. The spatial matching of measurements of different AFM techniques was satisfactory, based on the similarity between the potential and forward (−5 V) current images shown in Figure 4b and Figure 4c, as well as the topographies obtained (not shown here). The characterization was performed using only two values of the voltage (+5 V and −5 V), but a typical current-vs-voltage curve is shown in supplemental Figure S6 (Supporting Information File 1).

The correlated measurements and analysis of the potential and current are also applied to two different areas containing surface aggregates (Figure 4e,f for forward current and Figure 4g,h for reverse current). By correlating the potential and current measurements with one another, one can notice that the surface aggregates are defective with respect to current transport. Their defective nature is difficult to discern in the forward current image, although it is possible to observe small, sporadic non-conductive spots (dark blue) within the region of interest (black contour). On the other hand, the reverse current image shows almost entirely no current for the surface aggregates (white = no current). Because the aggregation layer blocks most of the reverse bias current and some of the forward bias current, charge transport through the aggregates from the active layer to top electrode could hinder PSC performance.

**Figure 5:**
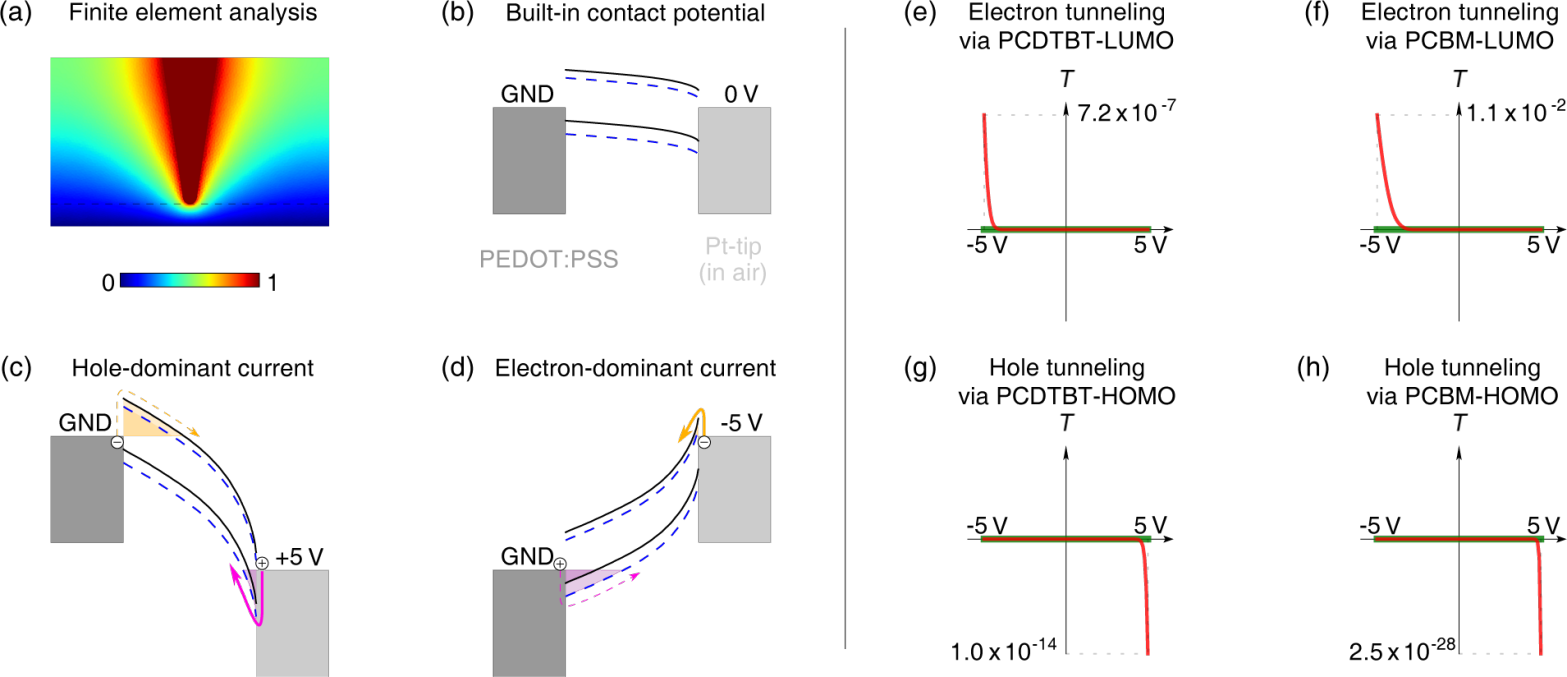
Finite element analysis and Wentzel–Kramers–Brillouin quantum tunneling calculations of the tip–electrode system and resulting energy band bending. (a) Electrostatic potential distribution in space between the tip (red) and bottom electrode. (b) Calculated energy diagram along the shortest path from the tip end to the bottom electrode showing the built-in contact potential difference and band bending. Solid black and dotted blue lines are used for the PCDTBT and PCBM energy levels, respectively. (c) Reverse bias current showing smaller barrier for holes at the tip side (pink arrow). (d) Forward bias current showing smaller barrier for electrons at the tip side (yellow arrow). (e) Transmission coefficient curves of electrons (red) injected at the tip side and flowing through the PCDTBT LUMO level to the bottom electrode, and holes (green, negligible) injected from the sample side flowing through the PCDTBT HOMO level to the tip. (f) Tunneling of electrons (red) injected from the tip side flowing through the PCBM LUMO level to the bottom electrode, and of holes (green, negligible) injected from the sample side flowing through the PCBM HOMO level to the tip. (g) Tunneling of holes (red) injected from the tip side flowing through the PCDTBT HOMO level to the bottom electrode, and of electrons (green, negligible) injected from the sample-side flowing through the PCDTBT LUMO level to the tip. (h) Tunneling of holes (red) injected from the tip side flowing through the PCBM HOMO level to the bottom electrode, and electrons (green, negligible) injected from the sample side flowing through the PCBM LUMO level to the tip. Note that in (e)–(h) the dominant carrier behavior is shown in red (and listed in the graph titles) and the minority carrier behavior is shown in green. Notice also the large differences in the scale of the vertical axes in (e)–(h).

In order to characterize the diode-like behavior under forward and reverse currents, we conducted three-dimensional finite element analysis (FEA) and charge tunneling calculations for the tip and bottom electrode system, as shown in Figure 5. The electric field distribution in AFM measurements differs from that of device-level measurements, which contain planar electrodes, such that a parallel-plate geometry analysis is appropriate there [28–29]. For the FEA simulation we used a tip radius of 25 nm, a tip cone angle of 17°, and a distance between tip and electrode of 80 nm. In addition, the typical energy levels of PCDTBT, PCBM and PEDOT:PSS were used to draw the energy diagrams in Figure 5b–d. Since the tip is exposed to air, we used 4.25 eV for the work function of platinum [30]. Because the tip diameter is several times smaller than the thickness of the active layer, the electric field is no longer homogeneous, but rather its strength increases near the tip (Figure 5a), which induces band bending towards the tip side (Figure 5b), resulting in a narrower energy barrier at the tip–sample junction (Figure 5c,d). Due to the band-bending effect and the moderate bias applied to the conductive tip, the charges are mainly injected at the tip side, compared with the bottom electrode of the PSC. This can be easily understood by comparing the energy barriers shown in Figure 5c,d (shaded area). The dominant charge carriers for the forward and reverse currents in our system are thus electrons and holes, respectively. The dominant charge carriers in C-AFM could be additionally optimized by choosing a more suitable tip coating material [31].

In addition to the qualitative energy-diagram analysis, we calculated the quantum tunneling transmission coefficient (*T*) of charge carriers through the barrier for the metal–donor and metal–acceptor interfaces. The transmission coefficient given by the Wentzel–Kramers–Brillouin (WKB) approximation has the form:


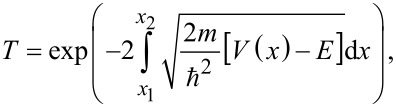


where *x*_1_ and *x*_2_ are the potential barrier boundaries, *m* is the mass of the charge carrier, 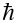 is the reduced Planck’s constant, *V*(*x*) is the bent energy level of either the lowest unoccupied molecular orbital (LUMO) or the highest occupied molecular orbital (HOMO) of PCDTBT and PCBM, and *E* is the biased Fermi energy of either the electron or hole at the Pt tip and the PEDOT:PSS. Since the measured currents in C-AFM are related to the transmission coefficient, it is useful to analyze all the possible tunneling routes as shown in Figure 5e–h. Red and green curves in the figures represent the tip-side and bottom-side injection of charge carriers, respectively. One can confirm that bottom-side injection of charges is much smaller than tip-side injection, as qualitatively discussed above. Additionally, the diode-like behavior of the metal–semiconductor junction is clearly discerned, which is consistent with the C-AFM measurements for the defective areas. The simulation results also show a difference of several orders of magnitude between the electron and hole transmission rate through the donor and acceptor materials. In accordance with the general working principle of PSCs, this means that the electron current prefers the acceptor pathway and the hole current prefers the donor pathway due to the energy differences of HOMO and LUMO levels. The calculations also indicate that the main charge carriers are injected from the tip-side, while opposite carrier injection from the bottom electrode is negligible. If one applies +5 V (−5 V) to the tip, then one is supplying holes (electrons) to the tip that are injected into the sample. At the same time, the corresponding injection of electrons (holes) from the bottom electrode is negligible. Therefore one mainly observes the flow of holes (electrons) upon application of +5 V (−5 V) to the tip.

According to the experimental and simulation results, the surface aggregates do not conduct holes, whereas they do not block much of the electron conduction. This is consistent with the observation of large potential drops in the area, if we consider that deficiency of holes (or accumulation of electrons) occurred in that area. Electrons generated from the active layer can be present anywhere, including the aggregates. However, generated holes cannot be present on the aggregates. The localization of negative charges in the surface aggregates leads to strong negative contrast in the KPFM measurements.

It is worth to noting here that the simulation results only depend on the energy levels of the materials and do not consider their spatial distribution. In real BHJ devices, both the macroscopic morphology and the microscopic conformation of donors and acceptors have a very strong influence on the overall mobility of charge carriers. Therefore, overlapping of HOMO and LUMO and charge hopping between molecules need to be considered for a proper fundamental understanding of the device physics and chemistry [32–34]. Nevertheless, the simulation provides useful qualitative insight for understanding C-AFM measurements on PSCs.

### Modification of surface aggregates by bimodal AFM

Since the surface aggregates are defective with regards to charge transport (entirely for holes and partially for electrons), revealing the underlying bulk active layer and observing the charge transport in the uncovered region is of interest. Although it is easy to remove the aggregates when scanning the defective area of an aged sample with KPFM, using aged samples is not ideal for this study due to the degradation of the active layer. The current signals, especially for the hole-dominant current, typically decrease by more than a factor of ten after several weeks of storage under ambient conditions. However, as mentioned above, the removal of aggregates with KPFM does not work for fresh samples. Thus, we apply controlled bimodal AFM indentation [35–36] to the surface aggregates of fresh samples in order to demonstrate the modification process. Since the aggregates are very thin, we vary the peak force of bimodal AFM just enough to break the aggregates, avoiding additional sample modification.

**Figure 6:**
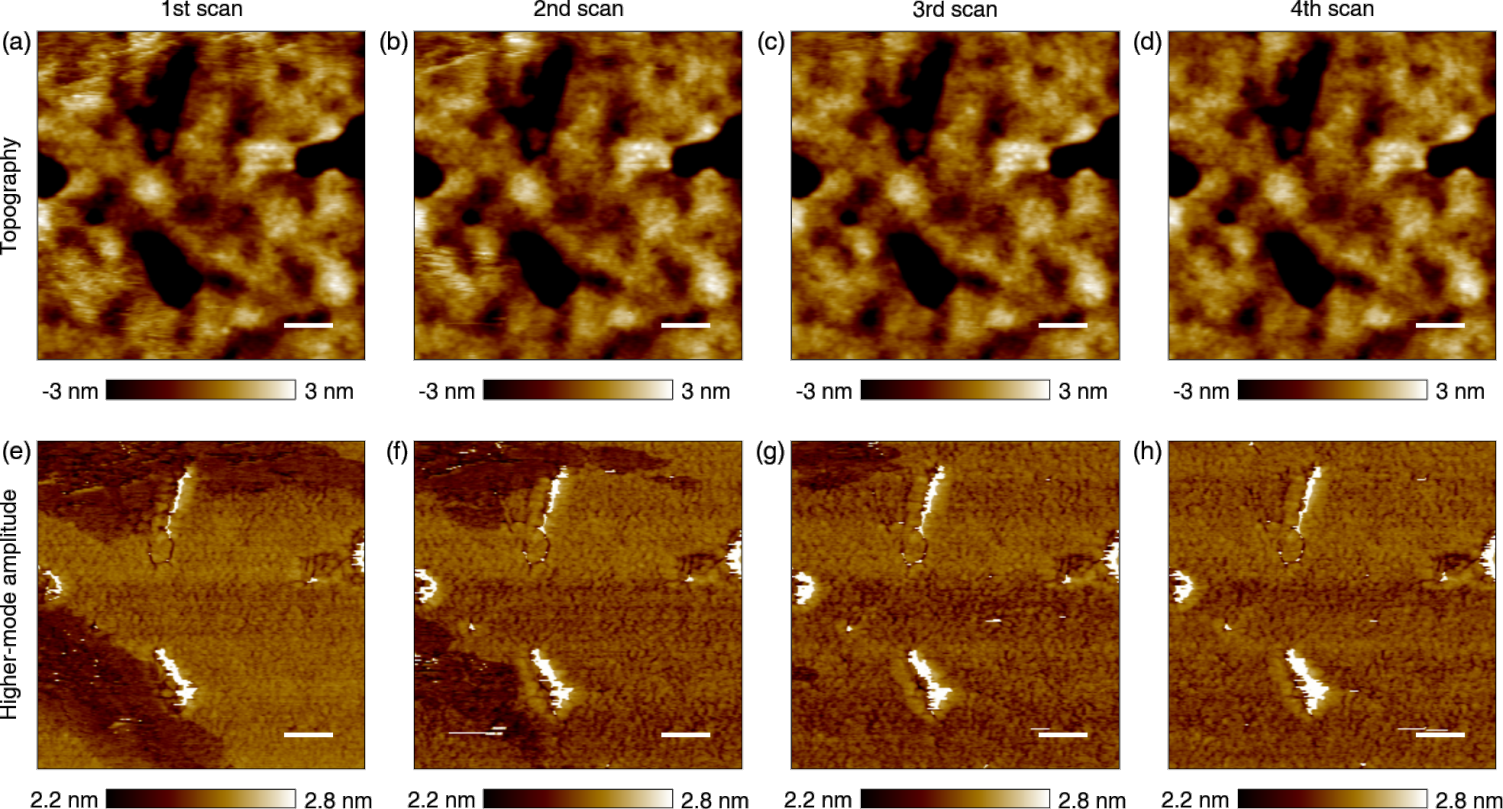
Removal of surface aggregates during consecutive bimodal AFM imaging. (a)–(d) Topographies consecutively obtained by tapping-mode bimodal AFM using the first eigenmode of the cantilever for the distance feedback control and the third eigenmode for modulating indentation. (e)–(h) The third eigenmode amplitude signal, corresponding to a free amplitude of about 3 nm, shows the aggregates as darker regions. The scale bars are 200 nm.

Typically, multifrequency AFM uses the first eigenmode of the cantilever to control the tip–sample distance and acquire the topography, and higher eigenmodes to measure additional properties [37–38]. We have also previously shown that the peak forces can be easily modulated by varying the amplitude of a higher eigenmode [36]. In our experiments, bimodal AFM using the first and third eigenmodes (approx. 65 kHz and approx. 1.2 MHz, respectively, Figure S7, Supporting Information File 1) of a Multi75E-G cantilever (Budget Sensors, Nanoworld) is implemented to break and remove the surface aggregates. As shown in Figure 6, four sets of images containing the topography and the third eigenmode amplitude are consecutively obtained with tapping-mode bimodal AFM. The surface aggregates are visualized as dark regions in the amplitude image and completely disappear within four consecutive image scans. One can also notice topographic variations in the regions corresponding to the darker sections of the amplitude images. Zig-zag dragging patterns disappear and the resulting final topography shows no significant evidence of the existence of aggregates. Because the spring constant of the third eigenmode is ca. 308 times the spring constant of the first mode [18], the indentation depth and peak force of the bimodal treatment are mainly controlled by the higher eigenmode [36]. The small free amplitude of the third eigenmode (ca. 3 nm, Figure S7, Supporting Information File 1) ensures controlled shallow indentation into the surface. Since the volume of aggregates in the scan area is about 0.58·10^−18^ L (with a total mass less than 1·10^−15^ g) [39], calculated from the dark region (36%) in Figure 6e and the thickness of aggregates previously obtained (ca. 0.8 nm), it is very difficult to determine the final destination of the material removed. We speculate that it is likely attached to the tip by attractive interactions such as van der Waals or chemical adhesion, following contact breaking and exfoliation.

**Figure 7:**
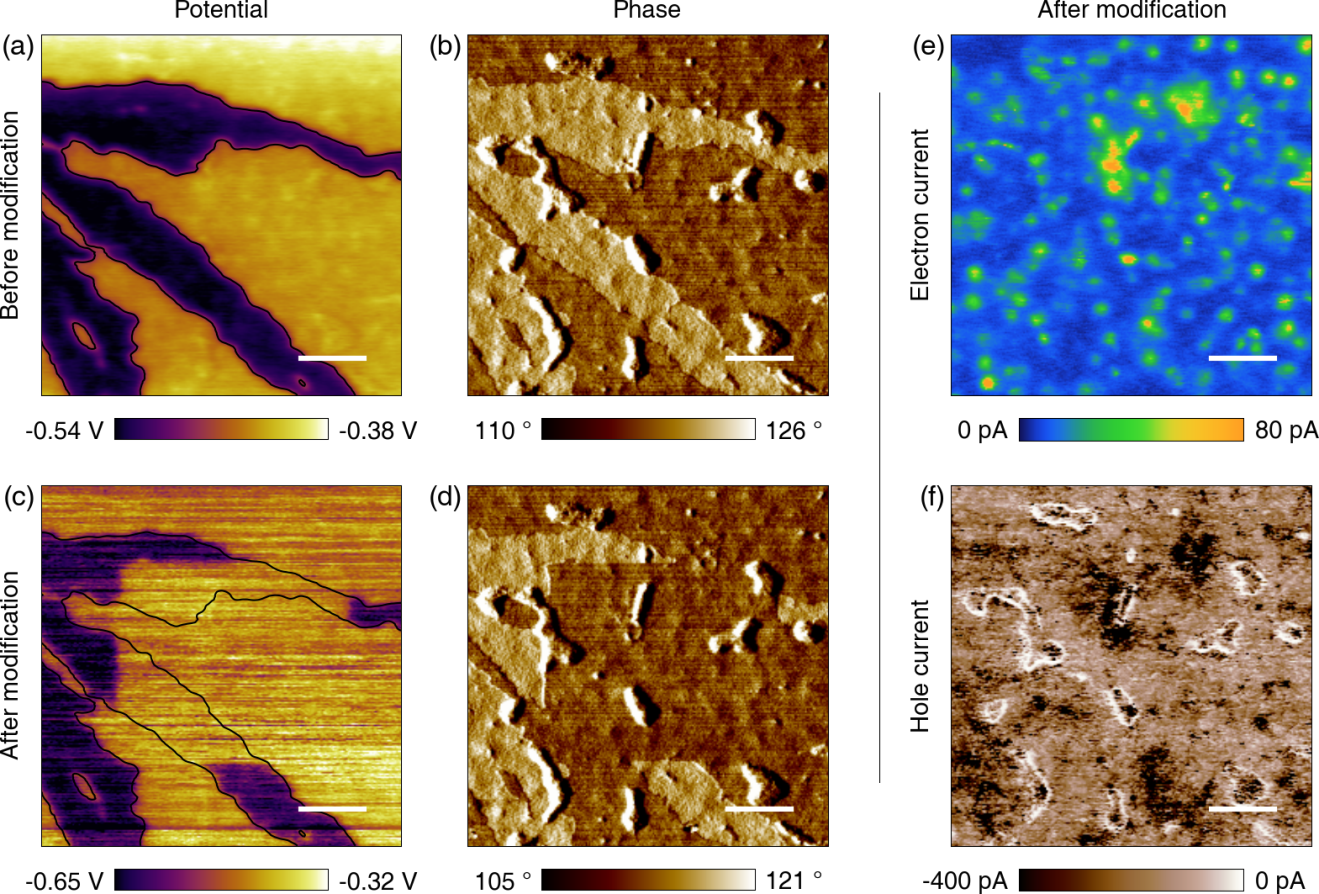
Potential and current measurements after removal of the surface aggregates. (a) Potential before the bimodal AFM treatment. (b) Phase before the treatment. (c) Potential after the bimodal AFM scans showing the treated central square region. (d) Corresponding phase after the bimodal scans. (e) Post-removal forward (electron) current showing no defective spots. (f) Post-removal reverse (hole) current showing no defective regions. Black contour lines highlight the defect boundaries in (a). The scale bars are 500 nm.

The effect of the bimodal AFM modification is observed in the potential and phase measurements (Figure 7a–d) before and after the treatment. The manipulated area is the central square region in Figure 7c,d. The potential and phase confirm that the surface aggregates disappeared and the underlying active layer is revealed, so that the area becomes similar to the ordinary surrounding area, i.e., the bulk active layer. Although the KPFM measurement shows removal of the aggregates, it is more instructive to observe the forward and reverse currents at the same area to verify the recovered functionality of the uncovered region. Here the measured currents (Figure 7e,f) show no defective behavior, thus demonstrating complete restoration of the active layer conductivity.

It is worth noting here that the current signals are recovered not only for the treated area but also for the area untreated with bimodal AFM (dark region in Figure 7c). This is because all of the surface aggregates disappeared during C-AFM imaging, due to the use of the stiffer cantilever Multi75E-G (Budget Sensors, force constant *k* ≈ 2 N/m, setpoint: 10–20 nN) to perform tapping-mode bimodal AFM sequentially with contact-mode C-AFM and noncontact-mode KPFM. The cantilever used for correlating the potential and currents in Figure 4 (PPP-CONTSCPt, NanoSensors) has a smaller spring constant (*k* = 0.5–1.0 N/m) and thus also a weaker torsional stiffness. In general, it is not possible to sustain a repulsive tapping-mode imaging process for the softer cantilever on polymer samples. On the other hand, the soft cantilever makes it possible to obtain current and potential correlations for the defective areas without severely altering the structure of the aggregates, by applying only a few nanonewtons of feedback force. Although it is in principle possible to use contact-mode AFM for the treatment, this is not as practical since the lateral friction force is not controlled, so that the contact-mode treatment sometimes leaves broken leftovers around the area of interest (see Figure S8, Supporting Information File 1) and can damage the sample.

### On the origin of the surface aggregates

The aggregates have been verified to be clusters of semiconducting molecular layers resting on top of the active layer. Since the structure showed defective behavior in transporting charge carriers, it is helpful to determine their origin. It is difficult to use techniques such as X-ray or electron diffraction spectroscopy due to the monolayer-like structure and the very small volume of the aggregates (of the order of one attoliter). We thus discuss the possible source of the aggregates, including donor polymer, acceptor polymer, other impurities and environmental contamination.

First, the samples were fabricated with the same method and procedure for the DCB and CB cases, thus we can exclude contamination from the environment. Second, the samples were fabricated many times over a period of several months using different batches of donor and acceptor bulk materials and showed consistently the same behavior, so that accidental addition of other impurities is not expected to be the source of the aggregates. Third, if donor polymer molecules accumulated at the surface, one would expect it to conduct holes (see Figure 5) and exhibit positive contrast for the potential measurement. However, if the aggregates are made of acceptor molecules, hole conduction would be deficient (see Figure 5) and localization of electrons would cause negative potential changes [40]. Furthermore, the measured thickness of the cluster layer is similar to the diameter of fullerenes if we consider partially buried PCBM molecules or molecules deformed by AFM indentation. Therefore, we conclude that the observed structures are most likely PCBM aggregates. PCBM aggregates in PCDTBT:PCBM PSCs cannot be discerned in bulk scale measurement for the normal annealing conditions used here [41]. However, they are found under typical fabrication conditions when the samples are examined at the nanoscale, such that sequential AFM measurements, as demonstrated here, can be extremely useful in further understanding and optimizing PSC performance.

## Conclusion

We report morphological and electrical transport property characterization of PCBM surface aggregates and modification of their structures on PCDTBT-based polymer solar cells using combined Kelvin probe (KPFM), conductive (C-AFM) and bimodal atomic force microscopy (AFM). Topography changes for partly removed aggregates show that the aggregates are composed of molecular thin layers of about 0.8 nm thickness. Correlated analysis of potential and current measurements shows that the aggregates exhibit defective charge transport, mainly for holes, with negative potential changes. The removal of the aggregates in a controlled way using bimodal AFM is performed to reveal the underlying active layer, so that forward and reverse currents are fully restored. Our systematic approach using combined KPFM, C-AFM and bimodal AFM in polymer solar cell studies can help elucidate unidentified structures at the nanoscale.

## Experimental

### Solution preparation

Anhydrous 99.8% chlorobenzene (Sigma-Aldrich, 284513) and 99% 1,2-dichlorobenzene (Sigma-Aldrich, 240664) solvents were used to dissolve PCDTBT (Sigma-Aldrich, 753998) and PCBM (Sigma-Aldrich, 684465). All materials and solvents were used as received. The weight ratio of PCDTBT and PCBM was 1:4, respectively, and the mixture concentration was 20 mg/mL for each solution. The solutions were stirred overnight on a hotplate at 80 °C. PCDTBT and PCBM were completely dissolved in dichlorobenzene solvent within a few hours and the solutions had no leftover. However, PCDTBT did not dissolve well into the chlorobenzene solvent, while PCBM did dissolve completely. There were PCDTBT leftovers precipitated on the wall and bottom of the solution container. The chlorobenzene solution containing dissolved and precipitated polymers was filtered through a 0.45 μm PTFE syringe filter to remove the precipitate. The dichlorobenzene solution was not filtered and was used as prepared.

### Device fabrication

Clean ITO-coated glass substrates (Sigma-Aldrich, 703192) were prepared by sonication with isopropyl alcohol and deionized water for 10 min, sequentially. A conductive polymer layer using high-conductivity grade 1.0 wt % in H_2_O PEDOT:PSS as received (Sigma-Aldrich, 768642) was spin-cast onto cleaned ITO substrates at 8,000 rpm, resulting in a film thickness of ca. 30 nm, and immediately dried at 150 °C for 10 min. An active layer was spin-cast onto the dried conductive layer producing a film of 70–80 nm thickness with either chlorobenzene (1,000 rpm) or dichlorobenzene (2,000 rpm) solution. Thicknesses of all layers were measured by AFM. The fabricated active layer and conductive layer were partly removed by wipes wetted with dichlorobenzene or DI-water wetted, to reveal the ITO substrate and make a contact later on. The samples were immediately dried at 70 °C for 30 min to evaporate the remaining solvent in the devices.

### AFM measurements

A series of AFM techniques including KPFM, C-AFM and bimodal AFM were combined to study the model PSCs. First, KPFM was used to find the surface aggregates and map surface potential changes due to the structures, then C-AFM was used to map local currents with positive and negative tip biases and correlate the currents with the surface potential. Afterwards, bimodal AFM was used to nanomechanically modify the aggregates. This was accomplished through the excitation of a higher cantilever eigenmode, which serves as a force-control “knob”, as recently reported [36]. To consecutively operate several AFM modes without changing hardware components, physical connections or cantilevers, a modified commercial AFM system (Asylum Research, MFP3D) was used. Multiple images using a combination of KPFM, C-AFM and bimodal AFM can be taken with a single cantilever on the same sample area, and they can be matched precisely and automatically by calculating cross-correlations of the topography images obtained (Figure S2, Supporting Information File 1). Typical conductive cantilevers, PPP-CONTSCPt (Nanosensors) and Multi75E-G (Budget Sensors), were used for the measurements of Figures 1–4 and Figures 6 and 7, respectively. All measurements were carried out under ambient conditions. For selected experiments the samples were illuminated from the bottom side with a white LED source, but the results did not display significant changes, except for a small offset in the measured potential between the dark and bright illumination conditions.

## Supporting Information

Supporting Information contains detailed experimental setup, image cross-correlation, analysis of an aged sample, time evolution of surface aggregates, aggregate removal during KPFM scans, a typical current–voltage curve, cantilever calibration, comparison of contact-mode and bimodal AFM treatments.

File 1 Additional experimental data.
