# Supplementary material for: Analysis and modification of defective surface aggregates on PCDTBT:PCBM solar cell blends using combined Kelvin probe, conductive and bimodal atomic force microscopy
Source: Beilstein J Nanotechnol. 2017 Mar 8;8:579–89. doi: 10.3762/bjnano.8.62 (PMC5355912; doi:10.3762/bjnano.8.62)
Supplement: File 1 — Additional experimental data. [file Beilstein_J_Nanotechnol-08-579-s001.pdf]

Supporting Information

for

Analysis and modification of defective surface aggregates on PCDTBT:PCBM solar cell blends using combined Kelvin probe, conductive and bimodal atomic force microscopy

Hanaul Noh, Alfredo J. Diaz and Santiago D. Solares*,[§]

Address: Department of Mechanical and Aerospace Engineering, The George Washington University, Washington, DC 20052, United States of America

Email: Santiago D. Solares* - ssolares@gwu.edu

* Corresponding author

[§] Phone: +1 (202) 994-0372

Additional experimental data

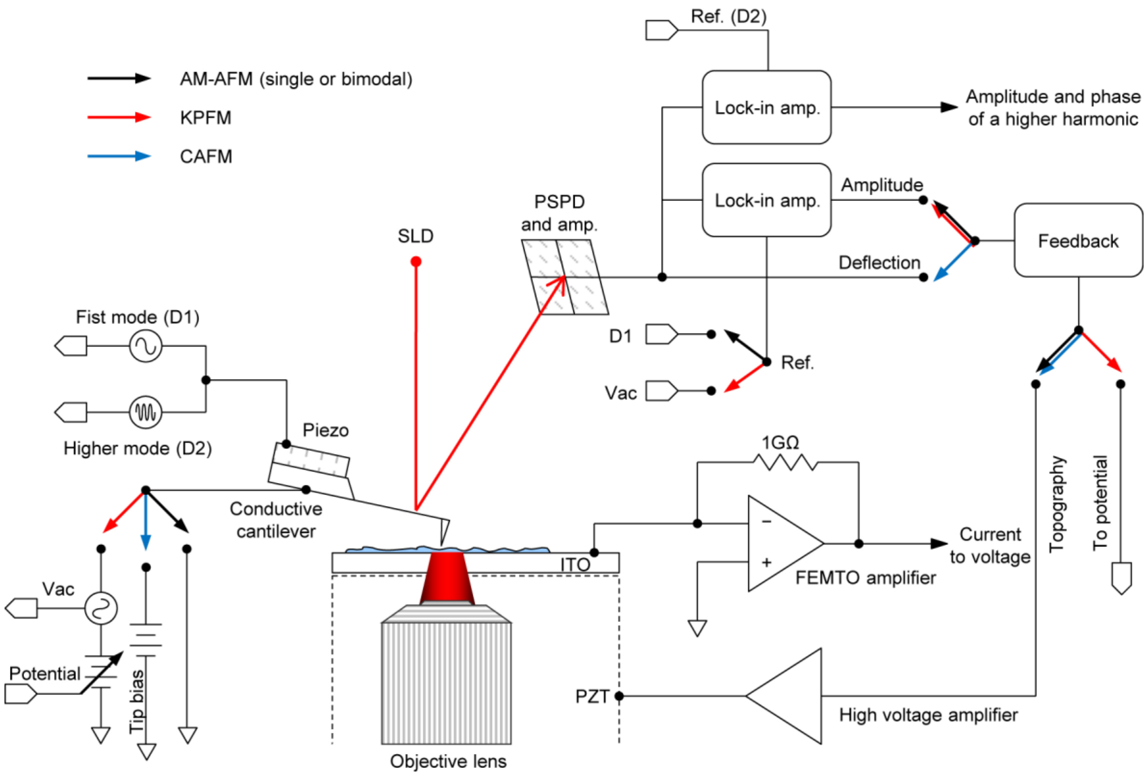

Figure S1: *Detailed experimental setup*. The schematic shows the full connections of the AFM, which enables performing KPFM, C-AFM and bimodal AFM on the same sample area without changing hardware components, physical connections or cantilevers. Any one of the AFM modes can be operated by configuring the electrical switches of a commercial AFM (Asylum Research, MFP3D) according to the colored arrows in the figure. A low-noise current amplifier (FEMTO®, DLPCA-200) is installed to have a better noise performance and to avoid replacement of the AFM cantilever holders. Because an external current amplifier is used, it is necessary to shield the power line noise sources for the current measurements. The shielding can be achieved by shortening the current path from the ITO contact to the input of the amplifier and covering the setup with a Faraday cage.

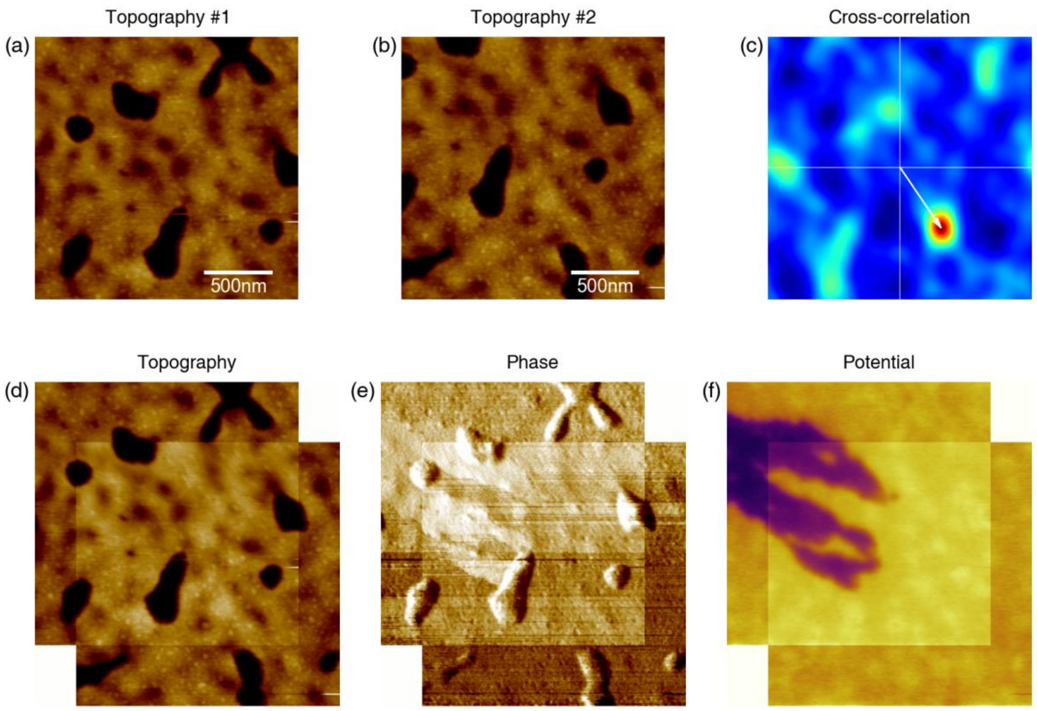

Figure S2: *Pattern matching of images by the cross-correlation method.* Since several measurements were performed sequentially at every sample position reported in the manuscript, correction of image drifts is very useful to analyze matching properties of topography, phase, potential and current. The correction was done by calculating the cross-correlation function of the topographic images. The cross-correlation of two functions is defined in real space as,

$$f \star g(\tau) = \int_{-\infty}^{\infty} f^*(t) g(t+\tau) dt.$$

It has the simple relation of $\mathcal{F}\{f \star g\} = \mathcal{F}(f)^* \cdot \mathcal{F}(g)$ in the frequency domain ($\mathcal{F}$: Fourier transform), similar to the convolution theorem. Thus, the cross-correlation of two topography images can be calculated very simply, as

$$C.C. = IFFT\{\, FFT(Topo1).conj \times FFT(Topo2)\, \}.$$

The maximum point in the cross-correlation image indicates the position deviation. By correcting the deviation, the signals can be automatically matched as shown in the figure.

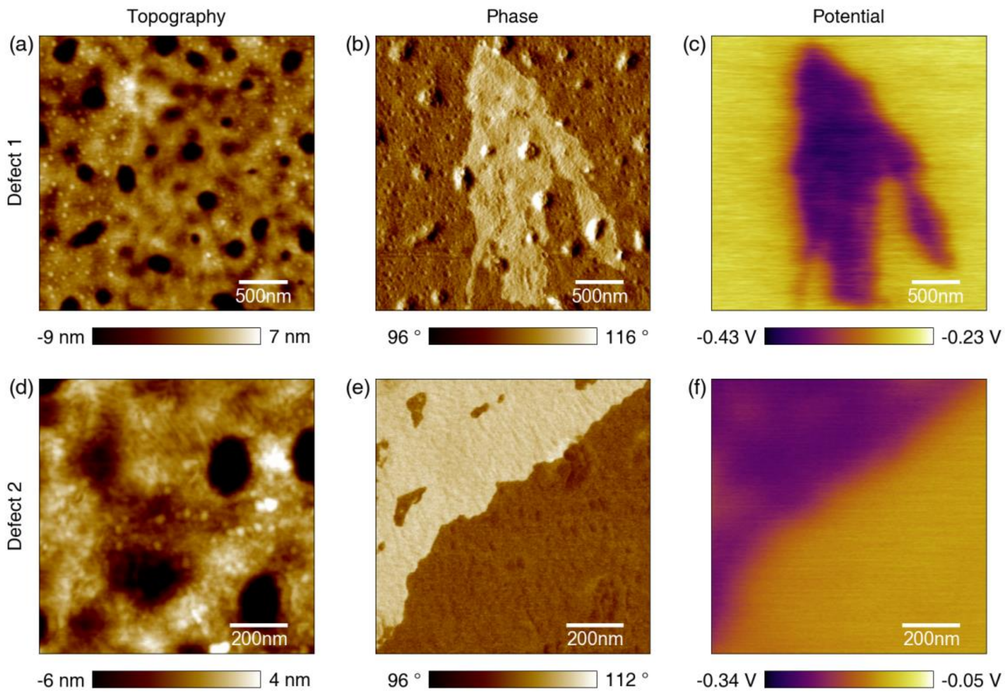

Figure S3: *Topography, phase and potential correspondence for an aged sample.* The measured topography shows variations corresponding to the phase and potential variations for the surface aggregates in an aged sample. In contrast, it is hard to find such topography correspondence in fresh samples. For the area of potential drop (aggregates), the topography does not exhibit small embossed dot-like structures and shows wrinkle-like structures as also shown in the phase images. The dot-like structures typically are not found in fresh samples but develop over time.

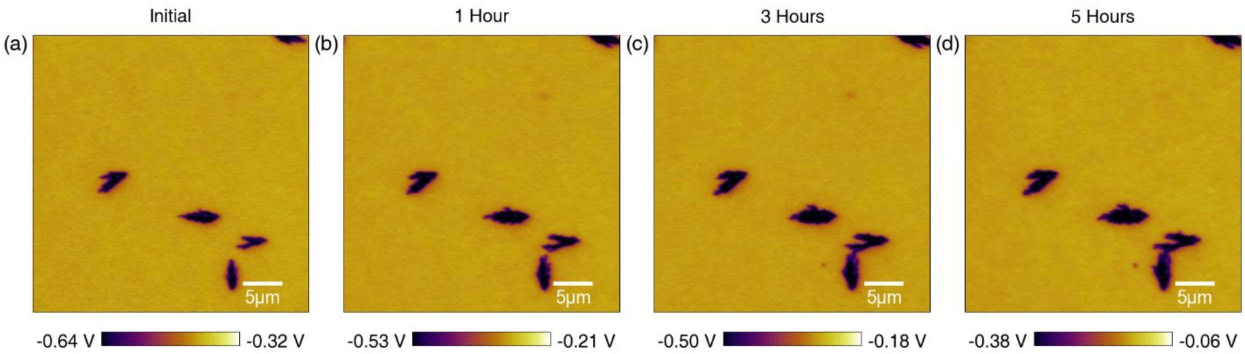

Figure S4: *Time evolution of the surface aggregates*. The aggregates expand over time and typically stop growing within several hours after fabrication. Although the mechanism of this process is unclear, this phenomenon could be related to either true aggregate growth or charging (localization) of electrons in the area.

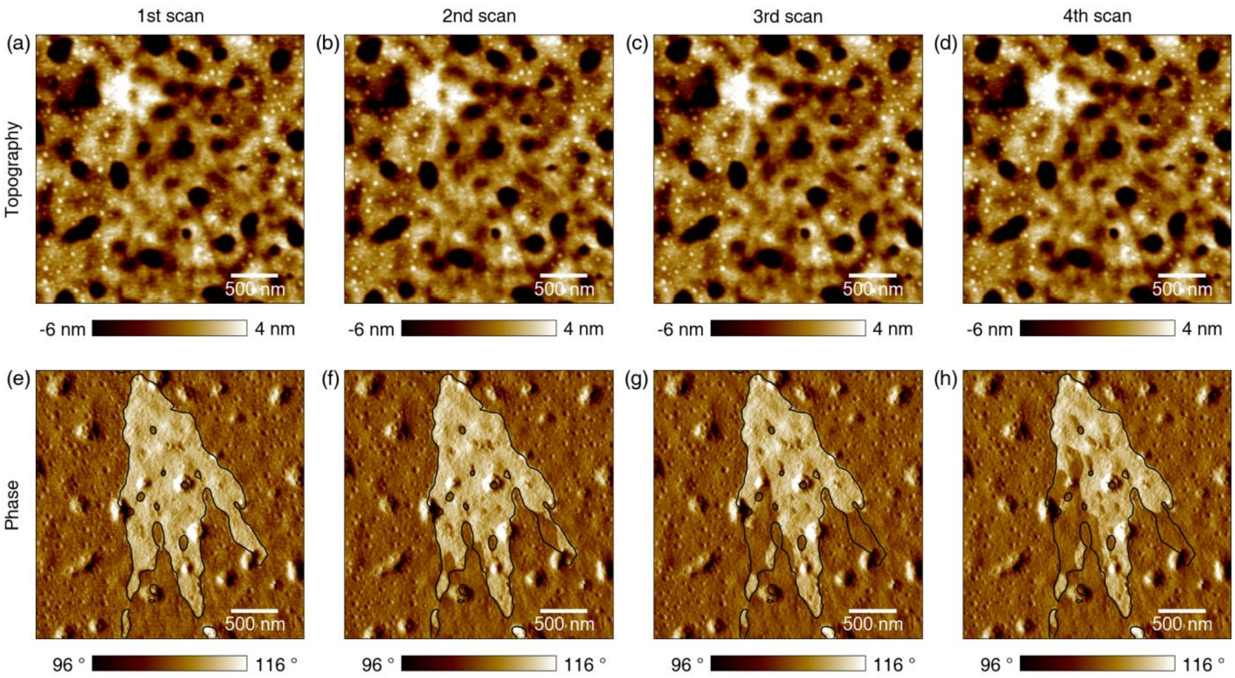

Figure S5: *Gradual removal of aggregates by consecutive KPFM scans*. As scanning progresses, the removal of aggregates advances piece by piece. Since the slow scans were done vertically, we conclude that the removal process is independent of the scanning direction. The black contour line highlights the initial boundary of the surface aggregates.

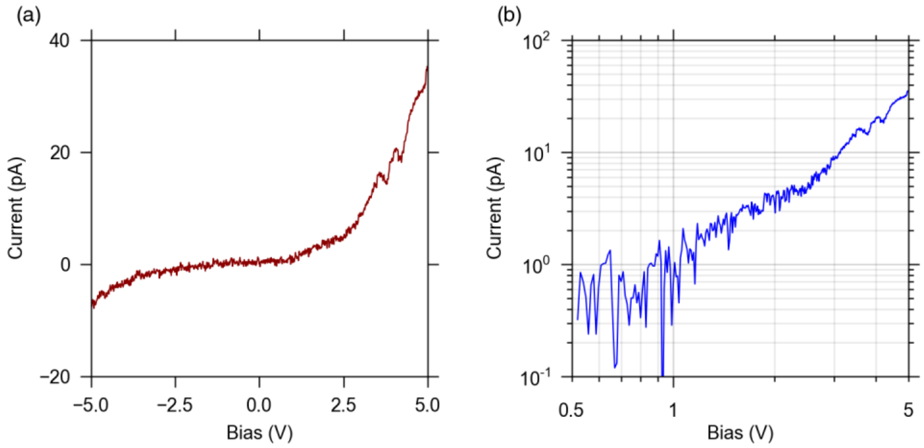

Figure S6: *A current vs. voltage curve with linear and log-log scales.* The current-voltage curve shown is only an example of a commonly observed shape. The shape of the curve can vary significantly as the sample contact position is changed.

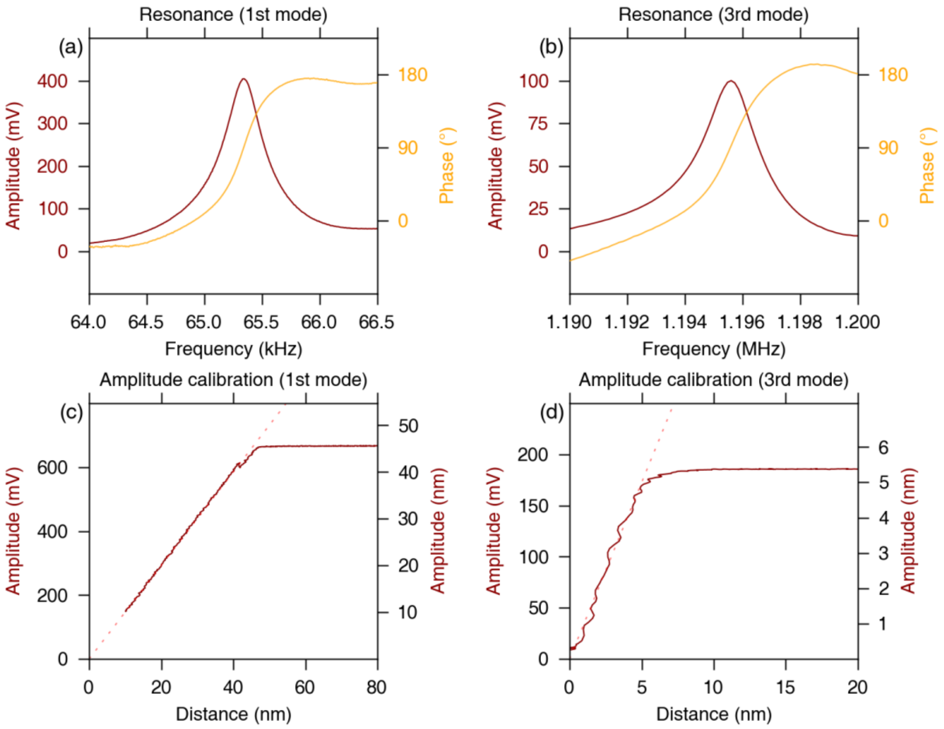

Figure S7: *Cantilever resonance and amplitude calibration curves.* The first and third eigenmodes of the Multi75E-G cantilever used for the bimodal manipulation are shown in (a) and (b), respectively. The oscillation amplitudes were calibrated by measuring amplitude-distance curves on a bare silicon substrate as shown in (c) and (d). The intermittent-contact regions of the curves were used for the linear fitting shown in (c) and (d) as dotted lines, giving calculated sensitivities of 68 nm/V and 29 nm/V for the first and third eigenmodes, respectively. The first eigenmode was optimized to maintain repulsive-regime tapping mode, and the third eigenmode was driven with a fixed *free* amplitude of 100 mV (this is an arbitrary value that depends on the setup), corresponding to 2.9 nm. The sensitivity parameters can vary for different cantilevers, imaging modes and AFM settings, such as detector laser spot position. Therefore, it is useful to calibrate the cantilever and know the real amplitude instead of using raw units, in order to perform controlled bimodal AFM.

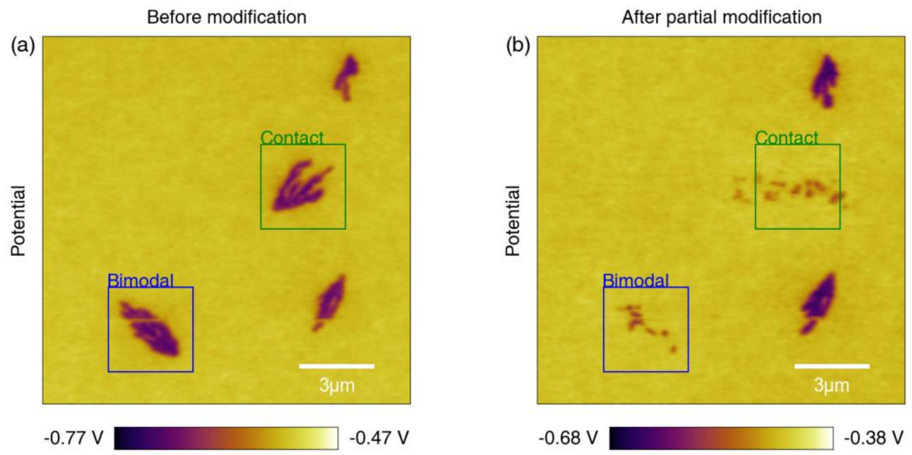

Figure S8: *Comparison between bimodal and contact mode modification of aggregates*. To compare the removal process of aggregates between the two AFM modes, partial modification of the target feature is applied. When we apply bimodal AFM to the area of interest, the modified region shows gradual removal of the aggregates, while portions of the aggregates remain at the original location. However, contact mode AFM destroys the structures and disperses the material away from the original position, as shown in the figure. The squares represent the scanning areas for each mode. The "over-scan" areas for the fast scan direction are not included in the squares.
